# Redetermination of methyl 3,4-*O*-isopropyl­idene-β-D-fucopyran­oside monohydrate

**DOI:** 10.1107/S1600536809012689

**Published:** 2009-04-08

**Authors:** Hoong-Kun Fun, Samuel Robinson Jebas, Sankappa Rai, Prakash Shetty, Arun M Isloor

**Affiliations:** a X-ray Crystallography Unit, School of Physics, Universiti Sains Malaysia, 11800 USM, Penang, Malaysia; b Syngene International Ltd, Biocon Park, Plot Nos. 2 & 3, Bommasandra 4th Phase, Jigani Link Rd, Bangalore 560 100, India; c Department of Printing, Manipal Institute of Technology, Manipal 576 104, India; d Department of Chemistry, National Institute of Technology-Karnataka, Surathkal, Mangalore 575 025, India

## Abstract

In the title compound, C_10_H_18_O_5_·H_2_O, the fucopyran­oside ring adopts a chair conformation. The crystal packing is stabilized by inter­molecular O—H⋯O and C—H⋯O hydrogen bonds together with intra­molecular O⋯O [2.2936 (8) Å] and inter­molecular O⋯O [2.7140 (8)–2.829 (3) Å] short contacts. The mol­ecules are linked together to form an infinite chain along the *a* axis. This structure has been solved previously but with no R-values [Spiers (1931). *Z. Kristallogr. Kristallgeom. Kristallphys. Kristallchem.* **78**, 101].

## Related literature

D-fucose (6-de­oxy-D-galactose) is an effective gratuitous inducer of the galactose operon in *Escherichia coli*, see: Musso *et al.* (1963 ▶). 6-Deoxy­hexose and its derivatives are important components of lipopolysaccharides, see: Bilge *et al.* (1996 ▶); Villeneuve *et al.* (2000 ▶); Wu & Mackenzie (1987 ▶); Caroff, Bundle & Perry (1984 ▶); Caroff, Bundle, Perry, Cherwonogrodzky & Dunch (1984 ▶). For a previous structure determination of the title compound, see: Spiers (1931 ▶). For bond-length data, see: Allen *et al.* (1987 ▶). For ring puckering analysis, see: Cremer & Pople (1975 ▶). For the stability of the temperature controller, see: Cosier & Glazer (1986 ▶).
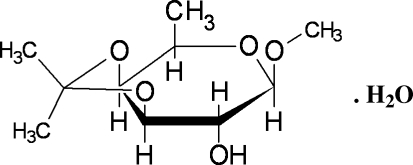


## Experimental

### 

#### Crystal data

C_10_H_18_O_5_·H_2_O
*M* *_r_* = 236.26
Orthorhombic, 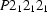

*a* = 8.5824 (1) Å
*b* = 9.2834 (1) Å
*c* = 14.6711 (2) Å
*V* = 1168.90 (2) Å^3^
*Z* = 4
Mo *K*α radiation
μ = 0.11 mm^−1^
*T* = 100 K
0.50 × 0.27 × 0.27 mm

#### Data collection

Bruker SMART APEXII CCD area-detector diffractometer
Absorption correction: multi-scan (**SADABS**; Bruker, 2005 ▶) *T*_min_ = 0.947, *T*_max_ = 0.971
64045 measured reflections
3449 independent reflections
3337 reflections with *I* > 2σ(*I*)
*R*_int_ = 0.032

#### Refinement

*R*[*F* ^2^ > 2σ(*F* ^2^)] = 0.026
*wR*(*F* ^2^) = 0.073
*S* = 1.13
3449 reflections
161 parameters
4 restraints
H atoms treated by a mixture of independent and constrained refinement
Δρ_max_ = 0.31 e Å^−3^
Δρ_min_ = −0.27 e Å^−3^

### 

Data collection: *APEX2* (Bruker, 2005 ▶); cell refinement: *SAINT* (Bruker, 2005 ▶); data reduction: *SAINT*; program(s) used to solve structure: *SHELXTL* (Sheldrick, 2008 ▶); program(s) used to refine structure: *SHELXTL*; molecular graphics: *SHELXTL*; software used to prepare material for publication: *SHELXTL* and *PLATON* (Spek, 2009 ▶).

## Supplementary Material

Crystal structure: contains datablocks global, I. DOI: 10.1107/S1600536809012689/is2405sup1.cif

Structure factors: contains datablocks I. DOI: 10.1107/S1600536809012689/is2405Isup2.hkl

Additional supplementary materials:  crystallographic information; 3D view; checkCIF report

## Figures and Tables

**Table 1:** Hydrogen-bond geometry (Å, °)

*D*—H⋯*A*	*D*—H	H⋯*A*	*D*⋯*A*	*D*—H⋯*A*
O4—H1*O*4⋯O1*W*^i^	0.821 (9)	1.909 (9)	2.7140 (8)	166.4 (16)
O1*W*—H1*W*1⋯O4^ii^	0.834 (8)	1.921 (8)	2.7534 (8)	175.6 (14)
O1*W*—H2*W*1⋯O5^iii^	0.838 (8)	2.113 (11)	2.8294 (8)	143.3 (15)
C9—H9*C*⋯O3^iv^	0.96	2.51	3.4306 (9)	162

Symmetry codes: (i) 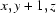; (ii) 
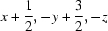
; (iii) 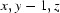; (iv) 
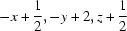
.

**Fig. 1.**
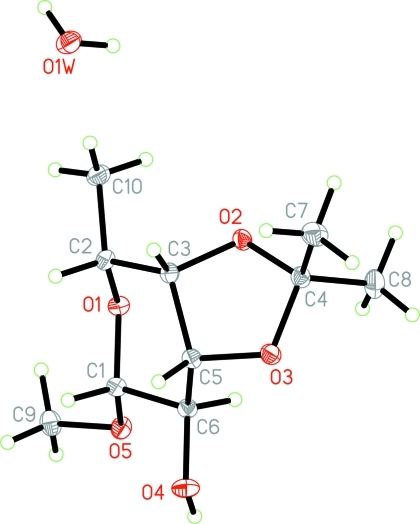
The molecular structure of the title compound, showing 50% probability displacement ellipsoids and the atom numbering scheme.

**Fig. 2.**
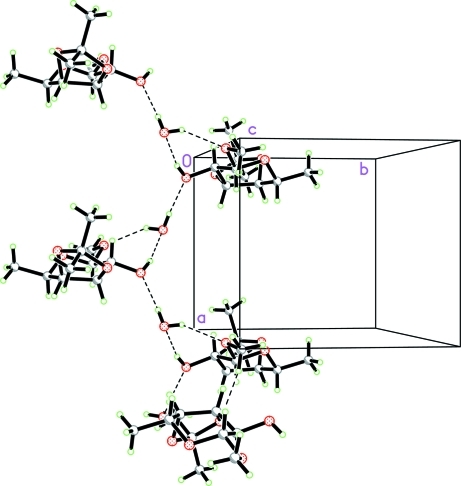
The crystal packing of the title compound, viewed approximately along the c axis, showing an extended molecular chain along the a axis. Dashed lines indicate the hydrogen bondings.

## supplementary crystallographic information

### Comment

Buttin has demonstrated that D-fucose (6-deoxy-D-galactose) is an effective gratuitous inducer of the galactose operon in *Escherichia coli* (Musso *et al.*, 1963). 6-Deoxyhexose and its derivatives are important components of lipopolysaccharides in, amongst others, *Vibrio cholerae* O1 (Bilge *et al.*, 1996), *Brucella* spc., *Citrobacter freundii* F90 (Villeneuve *et al.*, 2000), *Salmonella enterica* O30, and *Escherichia coli* O157 (Wu & Mackenzie, 1987). Further investigation revealed that D-fucose derivatives are important component of a repeating pentasaccharide unit in O-chains of the LPS of *Yersinia**enterocolitica* (Caroff, Bundle & Perry, 1984) and *Brucella abortus* (Caroff, Bundle, Perry, Cherwonogrodzky & Dunch, 1984). These findings established a molecular basis for extensive serological cross-reactivity between the various antigenic LPSs. These observations prompted us to synthesize the title compound, (I). Herein we report the synthesis and the redetermination of the crystal structure of the title compound.

The title compund has been determined previously (Spiers. 1931), but no *R*-values were given. The asymmetric unit of (I) (Fig.1) comprises of one molecule of methyl 3,4-*O*-isopropylidene -β-*D*–fucopyranoside and a water molecule. The isopropylidene-fucopyranoside ring is non-planar with the maximum deviation from planarity of 0.6532 (6) Å for the atom C5. The fucopyranoside ring adopts the chair conformation with the puckering parameters Q = 0.5344 (6), θ = 20.15 (7)° and φ = 22.3 (2)° (Cremer & Pople, 1975). The bond lengths (Allen *et al.*, 1987) and bond angles show normal values.

The crystal packing is stabilized by O—H···O and C—H···O hydrogen bonds to form infinite one dimensional chain along the [100] direction (Fig. 2). Short contacts of O···O = 2.2936 (8) Å; O···O^i^ = 2.7140 (8) Å; O···O^ii^ = 2.7535 (8) Å & O···O^iii^ = 2.8293 (8) Å [symmetry codes: (i) *x*,1 + *y*,*z*; (ii) -1/2 + *x*,3/2 - *y*,-*z* & (iii) *x*,1 + *y*,*z*] are observed.

### Experimental

The title compound was obtained by stirring a solution of 1,2,3,4 di-*O*-isopropylidene -α-*D*-fucopyranoside (0.5 g, 2.1 mmol) in dry methanol (5 ml). To this was added 3*M* solution of HCl in methanol (5 ml) at 0°C under nitrogen atmosphere. Further the reaction mixture was stirred at ambient temperature for 12 h. The reaction mixture was neutralized with solid sodium bicarbonate (1 g), concentrated, and residue was purified by flash column chromatography using 5% methanol in chloroform as eluent to get compound as foam-like solid which was taken in dry dimethylformamide (5 ml) and to this was added PTSA (Para Toluene Sulphonic Acid) (0.015 g, 2.0 mmol) and 2,2-dimethoxypropane (1.13 g,10 mmol). The mixture was further stirred at ambient temperature for 12 more hours. TLC (30% EtOAc/hexane,Rf-0.5) shows complete conversion of the starting materials. Reaction mixture was neutralized with triethylamine (2 ml) and concentrated under vacuum, residue was purified by column chromatography using 25% ethylacetate in pet ether to get a colourless liquid and the title compound as a white solid, which was recrystalized using hot acetone (yield 0.40 g, 85%; m.p. 328–330 K).

### Refinement

H atoms were positioned geometrically [C–H = 0.96–0.98 Å] and refined using a riding model, with *U*_iso_(H) = 1.2*U*_eq_(C) and 1.5*U*_eq_(methyl C). A rotating–group model was used for the methyl groups. The O-bound hydrogen atoms were located in a difference Fourier map and allowed to refine freely. Restraints were applied to fix the distance of O4—H = 0.82 (2) Å, O1W—H = 0.84 (2) Å and H1W1—H2W1 = 1.37 (4) Å. 2694 Friedel pairs were merged before final refinement as there is no large anomalous dispersion to determine the absolute configuration.

### Crystal data

**Table:**

C_10_H_18_O_5_·H_2_O	*F*(000) = 512
*M**_r_* = 236.26	*D*_x_ = 1.343 Mg m^−^^3^
Orthorhombic, *P*2_1_2_1_2_1_	Mo *K*α radiation, λ = 0.71073 Å
Hall symbol: P 2ac 2ab	Cell parameters from 9413 reflections
*a* = 8.5824 (1) Å	θ = 2.8–41.6°
*b* = 9.2834 (1) Å	µ = 0.11 mm^−^^1^
*c* = 14.6711 (2) Å	*T* = 100 K
*V* = 1168.90 (2) Å^3^	Block, colourless
*Z* = 4	0.50 × 0.27 × 0.27 mm

### Data collection

**Table:**

Bruker SMART APEXII CCD area-detector diffractometer	3449 independent reflections
Radiation source: fine-focus sealed tube	3337 reflections with *I* > 2σ(*I*)
graphite	*R*_int_ = 0.032
φ and ω scans	θ_max_ = 37.5°, θ_min_ = 2.6°
Absorption correction: multi-scan (*SADABS*; Bruker, 2005)	*h* = −14→14
*T*_min_ = 0.947, *T*_max_ = 0.971	*k* = −15→15
64045 measured reflections	*l* = −25→25

### Refinement

**Table:**

Refinement on *F*^2^	Primary atom site location: structure-invariant direct methods
Least-squares matrix: full	Secondary atom site location: difference Fourier map
*R*[*F*^2^ > 2σ(*F*^2^)] = 0.026	Hydrogen site location: inferred from neighbouring sites
*wR*(*F*^2^) = 0.073	H atoms treated by a mixture of independent and constrained refinement
*S* = 1.13	*w* = 1/[σ^2^(*F*_o_^2^) + (0.0439*P*)^2^ + 0.0731*P*] where *P* = (*F*_o_^2^ + 2*F*_c_^2^)/3
3449 reflections	(Δ/σ)_max_ = 0.001
161 parameters	Δρ_max_ = 0.31 e Å^−^^3^
4 restraints	Δρ_min_ = −0.27 e Å^−^^3^

### Special details

**Table:**

Experimental. The crystal was placed in the cold stream of an Oxford Cyrosystems Cobra open-flow nitrogen cryostat [Cosier, J. & Glazer, A. M. (1986). *J. Appl. Cryst.*19, 105–107] operating at 100.0 (1) K.
Geometry. All e.s.d.'s (except the e.s.d. in the dihedral angle between two l.s. planes) are estimated using the full covariance matrix. The cell e.s.d.'s are taken into account individually in the estimation of e.s.d.'s in distances, angles and torsion angles; correlations between e.s.d.'s in cell parameters are only used when they are defined by crystal symmetry. An approximate (isotropic) treatment of cell e.s.d.'s is used for estimating e.s.d.'s involving l.s. planes.
Refinement. Refinement of *F*^2^ against ALL reflections. The weighted *R*-factor *wR* and goodness of fit *S* are based on *F*^2^, conventional *R*-factors *R* are based on *F*, with *F* set to zero for negative *F*^2^. The threshold expression of *F*^2^ > σ(*F*^2^) is used only for calculating *R*-factors(gt) *etc*. and is not relevant to the choice of reflections for refinement. *R*-factors based on *F*^2^ are statistically about twice as large as those based on *F*, and *R*- factors based on ALL data will be even larger.

### Fractional atomic coordinates and isotropic or equivalent isotropic displacement parameters (Å^2^)

**Table:**

	*x*	*y*	*z*	*U*_iso_*/*U*_eq_	
O1	0.43844 (6)	0.76639 (5)	0.05482 (3)	0.01206 (8)	
O2	0.46299 (6)	0.72835 (6)	−0.14722 (3)	0.01333 (9)	
O3	0.37807 (7)	0.95475 (6)	−0.18528 (3)	0.01399 (9)	
O4	0.34767 (7)	1.13634 (6)	−0.01512 (4)	0.01634 (10)	
O5	0.48979 (6)	0.97492 (6)	0.13084 (3)	0.01364 (9)	
C1	0.38958 (8)	0.91139 (7)	0.06752 (4)	0.01172 (10)	
H1A	0.2818	0.9145	0.0895	0.014*	
C2	0.33053 (8)	0.68941 (7)	−0.00168 (4)	0.01206 (10)	
H2A	0.2268	0.6964	0.0259	0.014*	
C3	0.32312 (8)	0.75401 (7)	−0.09665 (4)	0.01172 (10)	
H3A	0.2342	0.7132	−0.1296	0.014*	
C4	0.46232 (8)	0.83123 (7)	−0.21980 (4)	0.01328 (10)	
C5	0.31314 (7)	0.91860 (7)	−0.09849 (4)	0.01160 (10)	
H5A	0.2038	0.9487	−0.0961	0.014*	
C6	0.40456 (8)	0.99275 (7)	−0.02236 (4)	0.01164 (10)	
H6A	0.5148	0.9961	−0.0396	0.014*	
C7	0.37798 (10)	0.77431 (9)	−0.30350 (5)	0.01941 (13)	
H7A	0.2738	0.7469	−0.2871	0.029*	
H7B	0.4326	0.6920	−0.3269	0.029*	
H7C	0.3742	0.8481	−0.3493	0.029*	
C8	0.62911 (9)	0.87289 (9)	−0.24007 (5)	0.01995 (13)	
H8A	0.6795	0.9026	−0.1848	0.030*	
H8B	0.6305	0.9508	−0.2831	0.030*	
H8C	0.6833	0.7917	−0.2653	0.030*	
C9	0.46504 (9)	0.92419 (8)	0.22239 (4)	0.01716 (12)	
H9A	0.5330	0.9749	0.2633	0.026*	
H9B	0.4870	0.8229	0.2253	0.026*	
H9C	0.3587	0.9409	0.2396	0.026*	
C10	0.37923 (9)	0.53235 (7)	−0.00202 (5)	0.01627 (11)	
H10A	0.3747	0.4950	0.0589	0.024*	
H10B	0.4838	0.5243	−0.0247	0.024*	
H10C	0.3101	0.4784	−0.0405	0.024*	
O1W	0.57299 (7)	0.26179 (6)	0.08757 (4)	0.01852 (10)	
H1O4	0.4072 (16)	1.1865 (15)	0.0151 (9)	0.032 (4)*	
H1W1	0.6579 (13)	0.2881 (14)	0.0656 (9)	0.030 (4)*	
H2W1	0.5836 (19)	0.1840 (12)	0.1158 (10)	0.041 (4)*	

### Atomic displacement parameters (Å^2^)

**Table:**

	*U*^11^	*U*^22^	*U*^33^	*U*^12^	*U*^13^	*U*^23^
O1	0.01237 (19)	0.01127 (17)	0.01255 (18)	0.00025 (15)	−0.00175 (15)	0.00011 (15)
O2	0.01453 (19)	0.01320 (18)	0.01227 (18)	0.00295 (16)	0.00272 (15)	0.00221 (15)
O3	0.0180 (2)	0.01283 (19)	0.01116 (18)	0.00330 (17)	0.00186 (16)	0.00176 (15)
O4	0.0190 (2)	0.01123 (18)	0.0188 (2)	0.00316 (17)	−0.00409 (18)	−0.00123 (17)
O5	0.0155 (2)	0.0152 (2)	0.01031 (18)	−0.00255 (16)	−0.00114 (15)	−0.00001 (15)
C1	0.0116 (2)	0.0122 (2)	0.0114 (2)	0.00024 (19)	−0.00052 (18)	−0.00035 (18)
C2	0.0118 (2)	0.0122 (2)	0.0122 (2)	−0.00124 (18)	−0.00016 (19)	0.00084 (18)
C3	0.0112 (2)	0.0126 (2)	0.0114 (2)	−0.00007 (18)	−0.00006 (18)	0.00028 (19)
C4	0.0146 (2)	0.0138 (2)	0.0114 (2)	0.0020 (2)	0.00107 (19)	0.00140 (19)
C5	0.0111 (2)	0.0127 (2)	0.0110 (2)	0.00160 (18)	−0.00033 (18)	0.00072 (18)
C6	0.0119 (2)	0.0111 (2)	0.0119 (2)	0.00140 (17)	−0.00036 (18)	0.00060 (18)
C7	0.0262 (3)	0.0196 (3)	0.0124 (2)	0.0016 (3)	−0.0019 (2)	−0.0015 (2)
C8	0.0159 (3)	0.0246 (3)	0.0193 (3)	0.0013 (2)	0.0040 (2)	0.0059 (3)
C9	0.0185 (3)	0.0222 (3)	0.0107 (2)	−0.0003 (2)	0.0004 (2)	0.0003 (2)
C10	0.0199 (3)	0.0121 (2)	0.0169 (3)	−0.0007 (2)	−0.0002 (2)	0.0010 (2)
O1W	0.0186 (2)	0.0147 (2)	0.0223 (2)	−0.00180 (18)	−0.00014 (19)	0.00053 (19)

### Geometric parameters (Å, °)

**Table:**

O1—C1	1.4222 (8)	C4—C7	1.5202 (10)
O1—C2	1.4336 (8)	C5—C6	1.5287 (9)
O2—C4	1.4304 (8)	C5—H5A	0.9800
O2—C3	1.4311 (8)	C6—H6A	0.9800
O3—C5	1.4299 (8)	C7—H7A	0.9600
O3—C4	1.4472 (8)	C7—H7B	0.9600
O4—C6	1.4236 (8)	C7—H7C	0.9600
O4—H1O4	0.821 (9)	C8—H8A	0.9600
O5—C1	1.3966 (8)	C8—H8B	0.9600
O5—C9	1.4389 (8)	C8—H8C	0.9600
C1—C6	1.5251 (9)	C9—H9A	0.9600
C1—H1A	0.9800	C9—H9B	0.9600
C2—C10	1.5168 (10)	C9—H9C	0.9600
C2—C3	1.5183 (9)	C10—H10A	0.9600
C2—H2A	0.9800	C10—H10B	0.9600
C3—C5	1.5306 (9)	C10—H10C	0.9600
C3—H3A	0.9800	O1W—H1W1	0.834 (8)
C4—C8	1.5123 (10)	O1W—H2W1	0.838 (8)
			
C1—O1—C2	110.92 (5)	C6—C5—H5A	109.7
C4—O2—C3	105.75 (5)	C3—C5—H5A	109.7
C5—O3—C4	108.68 (5)	O4—C6—C1	111.74 (5)
C6—O4—H1O4	111.0 (12)	O4—C6—C5	107.46 (5)
C1—O5—C9	113.06 (5)	C1—C6—C5	111.43 (5)
O5—C1—O1	107.78 (5)	O4—C6—H6A	108.7
O5—C1—C6	108.30 (5)	C1—C6—H6A	108.7
O1—C1—C6	109.30 (5)	C5—C6—H6A	108.7
O5—C1—H1A	110.5	C4—C7—H7A	109.5
O1—C1—H1A	110.5	C4—C7—H7B	109.5
C6—C1—H1A	110.5	H7A—C7—H7B	109.5
O1—C2—C10	107.64 (5)	C4—C7—H7C	109.5
O1—C2—C3	111.14 (5)	H7A—C7—H7C	109.5
C10—C2—C3	112.84 (6)	H7B—C7—H7C	109.5
O1—C2—H2A	108.4	C4—C8—H8A	109.5
C10—C2—H2A	108.4	C4—C8—H8B	109.5
C3—C2—H2A	108.4	H8A—C8—H8B	109.5
O2—C3—C2	112.02 (5)	C4—C8—H8C	109.5
O2—C3—C5	101.77 (5)	H8A—C8—H8C	109.5
C2—C3—C5	114.38 (5)	H8B—C8—H8C	109.5
O2—C3—H3A	109.5	O5—C9—H9A	109.5
C2—C3—H3A	109.5	O5—C9—H9B	109.5
C5—C3—H3A	109.5	H9A—C9—H9B	109.5
O2—C4—O3	105.70 (5)	O5—C9—H9C	109.5
O2—C4—C8	108.26 (6)	H9A—C9—H9C	109.5
O3—C4—C8	109.83 (6)	H9B—C9—H9C	109.5
O2—C4—C7	111.79 (6)	C2—C10—H10A	109.5
O3—C4—C7	108.67 (6)	C2—C10—H10B	109.5
C8—C4—C7	112.38 (6)	H10A—C10—H10B	109.5
O3—C5—C6	110.18 (5)	C2—C10—H10C	109.5
O3—C5—C3	103.19 (5)	H10A—C10—H10C	109.5
C6—C5—C3	114.08 (5)	H10B—C10—H10C	109.5
O3—C5—H5A	109.7	H1W1—O1W—H2W1	110.4 (12)
			
C9—O5—C1—O1	−72.50 (7)	C5—O3—C4—C8	−123.77 (6)
C9—O5—C1—C6	169.36 (6)	C5—O3—C4—C7	112.93 (6)
C2—O1—C1—O5	173.54 (5)	C4—O3—C5—C6	106.03 (6)
C2—O1—C1—C6	−68.96 (6)	C4—O3—C5—C3	−16.15 (7)
C1—O1—C2—C10	−172.91 (5)	O2—C3—C5—O3	33.37 (6)
C1—O1—C2—C3	63.06 (7)	C2—C3—C5—O3	154.36 (5)
C4—O2—C3—C2	−161.33 (5)	O2—C3—C5—C6	−86.15 (6)
C4—O2—C3—C5	−38.71 (6)	C2—C3—C5—C6	34.84 (8)
O1—C2—C3—O2	70.09 (7)	O5—C1—C6—O4	−66.61 (7)
C10—C2—C3—O2	−50.94 (7)	O1—C1—C6—O4	176.22 (5)
O1—C2—C3—C5	−45.05 (8)	O5—C1—C6—C5	173.15 (5)
C10—C2—C3—C5	−166.08 (6)	O1—C1—C6—C5	55.99 (7)
C3—O2—C4—O3	29.57 (7)	O3—C5—C6—O4	82.09 (6)
C3—O2—C4—C8	147.20 (6)	C3—C5—C6—O4	−162.42 (5)
C3—O2—C4—C7	−88.49 (7)	O3—C5—C6—C1	−155.19 (5)
C5—O3—C4—O2	−7.19 (7)	C3—C5—C6—C1	−39.69 (8)

### Hydrogen-bond geometry (Å, °)

**Table:**

*D*—H···*A*	*D*—H	H···*A*	*D*···*A*	*D*—H···*A*
O4—H1O4···O1W^i^	0.82 (1)	1.91 (1)	2.7140 (8)	166 (2)
O1W—H1W1···O4^ii^	0.83 (1)	1.92 (1)	2.7534 (8)	176 (1)
O1W—H2W1···O5^iii^	0.84 (1)	2.11 (1)	2.8294 (8)	143 (2)
C9—H9C···O3^iv^	0.96	2.51	3.4306 (9)	162

Symmetry codes: (i) *x*, *y*+1, *z*; (ii) *x*+1/2, −*y*+3/2, −*z*; (iii) *x*, *y*−1, *z*; (iv) −*x*+1/2, −*y*+2, *z*+1/2.
